# Effects of x-ray free-electron laser pulse intensity on the Mn K***β***_1,3_ x-ray emission spectrum in photosystem II—A case study for metalloprotein crystals and solutions

**DOI:** 10.1063/4.0000130

**Published:** 2021-11-22

**Authors:** Thomas Fransson, Roberto Alonso-Mori, Ruchira Chatterjee, Mun Hon Cheah, Mohamed Ibrahim, Rana Hussein, Miao Zhang, Franklin Fuller, Sheraz Gul, In-Sik Kim, Philipp S. Simon, Isabel Bogacz, Hiroki Makita, Casper de Lichtenberg, Sanghoon Song, Alexander Batyuk, Dimosthenis Sokaras, Ramzi Massad, Margaret Doyle, Alexander Britz, Clemens Weninger, Athina Zouni, Johannes Messinger, Vittal K. Yachandra, Junko Yano, Jan Kern, Uwe Bergmann

**Affiliations:** 1 Department of Theoretical Chemistry and Biology, KTH Royal Institute of Technology, Stockholm, Sweden; 2 Linac Coherent Light Source, SLAC National Accelerator Laboratory, Menlo Park, California 94025, USA; 3 Molecular Biophysics and Integrated Bioimaging Division, Lawrence Berkeley National Laboratory, Berkeley, California 94720, USA; 4 Department of Chemistry – Ångström Laboratory, Molecular Biomimetics, Uppsala University, SE 75120 Uppsala, Sweden; 5 Humboldt-Universität zu Berlin, Department of Biology, 10099 Berlin, Germany; 6 Department of Chemistry, Umeå University, SE 90187 Umeå, Sweden; 7 Section of Biomolecular Sciences, Department of Biology, University of Copenhagen, 2200 Copenhagen N, Denmark; 8 Stanford Synchrotron Radiation Lightsource, SLAC National Accelerator Laboratory, Menlo Park, California 94025, USA; 9 Stanford PULSE Institute, SLAC National Accelerator Laboratory, Menlo Park, California 94025, USA; 10 MAX IV Laboratory, Lund University, Lund, Sweden; 11 Department of Physics, University of Wisconsin-Madison, Madison, Wisconsin 53706, USA

## Abstract

In the last ten years, x-ray free-electron lasers (XFELs) have been successfully employed to characterize metalloproteins at room temperature using various techniques including x-ray diffraction, scattering, and spectroscopy. The approach has been to outrun the radiation damage by using femtosecond (fs) x-ray pulses. An example of an important and damage sensitive active metal center is the Mn_4_CaO_5_ cluster in photosystem II (PS II), the catalytic site of photosynthetic water oxidation. The combination of serial femtosecond x-ray crystallography and Kβ x-ray emission spectroscopy (XES) has proven to be a powerful multimodal approach for simultaneously probing the overall protein structure and the electronic state of the Mn_4_CaO_5_ cluster throughout the catalytic (Kok) cycle. As the observed spectral changes in the Mn_4_CaO_5_ cluster are very subtle, it is critical to consider the potential effects of the intense XFEL pulses on the Kβ XES signal. We report here a systematic study of the effects of XFEL peak power, beam focus, and dose on the Mn Kβ_1,3_ XES spectra in PS II over a wide range of pulse parameters collected over seven different experimental runs using both microcrystal and solution PS II samples. Our findings show that for beam intensities ranging from ∼5 × 10^15^ to 5 × 10^17^ W/cm^2^ at a pulse length of ∼35 fs, the spectral effects are small compared to those observed between S-states in the Kok cycle. Our results provide a benchmark for other XFEL-based XES studies on metalloproteins, confirming the viability of this approach.

## I. INTRODUCTION

X-ray spectroscopy is a powerful element-sensitive technique for probing the local structure of the active site in metalloproteins and model inorganic complexes.^1–9^ In metalloproteins, it has been widely used for the mechanistic understanding of the metal catalytic centers using x-rays at synchrotron facilities. The oxygen evolving complex (OEC) in Photosystem II (PS II) is one of such systems, consisting of an oxo-bridged tetra-manganese calcium (Mn_4_CaO_5_) catalytic site in which photosynthetic water oxidation is carried out.^10–13^ During the water oxidation reaction, the OEC cycles between several oxidation states with the S_3_ state reached after two light flashes (2F) being the most oxidized stable state [with Mn(IV)_4_], whereas the dark stable S_1_ state (0F) has an oxidation state of Mn(III)_2_(IV)_2_. One of the main challenges in the several decades of synchrotron-based x-ray spectroscopy and diffraction studies of the OEC has been the modification of the geometric and the electronic structures caused by Mn metal reduction and protein modification due to the synchrotron x-ray beams. These synchrotron radiation-induced damage processes are related to migration of radicals and other relatively slow processes in the range of ∼20 ps.^14,15^ For the synchrotron study of the Mn K edge with x-ray absorption spectroscopy (XAS), x-ray emission spectroscopy (XES), and resonant inelastic x-ray scattering (RIXS) in the 6–10 keV range, this problem has been addressed by (a) cryogenic cooling of the sample, and (b) frequently moving the beam to a new sample position to minimize the dose. The seminal study of x-ray induced damage to the Mn_4_CaO_5_ cluster by Yano *et al.*^16^ established the fraction of photoreduced Mn(II) species using x-ray absorption near-edge structure (XANES) and the destruction of the metal cluster structure using extended x-ray absorption fine structure (EXAFS) as a function of x-ray energy, dose, and sample temperature. It also showed that essentially all x-ray diffraction studies to determine the protein structure of PS II crystals until 2005 had used doses where the OEC was strongly photo reduced and its geometric and electronic structure strongly modified. Showing the large increase in x-ray damage with sample temperature, the study further suggested that synchrotron-based room temperature studies of the OEC would be extremely challenging, and very few such studies have been reported.^17,18^ The effect is even much more severe at soft x-ray energies. This is due to the combination of a large absorption cross section, larger Auger electron yield, and the corresponding small fluorescence yield in the soft x-ray region, essentially preventing a rapid enough sample replacement. Despite decades of efforts, the problem of radiation damage has not been fully solved for synchrotron-based soft x-ray studies of the Mn L-edge (635–655 eV) of the OEC.^19^

This situation dramatically changed with the advent of the x-ray free-electron laser (XFEL), where extremely intense femtosecond x-ray pulses are employed.^20–22^ XFEL pulses are so short (∼5–100 fs) and so widely spaced in time [∼8 ms at the Linac Coherent Light Source (LCLS)], that (a) the x-ray probe can potentially outrun the sample damage, and (b) the entire sample can be replaced before the next pulse arrives. Neutze *et al.* were the first to theoretically estimate the timescale of the Coulomb explosion of a protein molecule when exposed to an intense XFEL pulse.^23^ Their original suggestion that it should be possible to outrun the damage has been confirmed experimentally many times, and this so-called “probe-before-destroy” approach has become a critical tool for much of the XFEL-based research. This method has been used for single particle imaging,^24,25^ in serial femtosecond crystallography,^26–29^ coherent diffractive imaging,^30^ x-ray spectroscopy^5,8,31–35^ and x-ray sppectroscopy combined with diffraction/scattering experiments.^36–39^ The probe-before-destroy approach made it possible to study the atomic and Mn electronic structure of PS II at room temperature in the four metastable intermediate states (S_0_, S_1_, S_2_, and S_3_) of the Kok cycle^36,37,40–46^ and time points during transition between these states.^40,45^ The Mn oxidation states throughout the metastable intermediates vary from [III_3_,IV] (S_0_) to [IV_4_] (S_3_), with the potential of a further oxidized S_4_ state occurring just before O_2_ formation. The results from these XFEL-based studies have significantly advanced our understanding of the water oxidation mechanism.

Starting with the work by Alonso-Mori *et al.* and Kern *et al.*, it was established that XFEL-based Kβ XES spectra of chemical compounds^31^ and the OEC^36^ collected at room temperature show essentially no beam-induced effects on the Mn electronic structure. This is remarkable, as the x-ray dose under these XFEL pulse conditions with 10^17^–10^18^ W cm^−2^ peak power corresponds to 10^6^–10^8^ Gray depending on the beam size used [∼2.5–10 *μ*m, FWHM (full width half maximum)]. This is up to ten times higher than the dose reported by Yano *et al.* using synchrotron radiation,^16^ at which more than 90% of the Mn atoms of the Mn_4_CaO_5_ cluster are reduced to Mn(II) at 100 K temperature, and with the metal cluster being entirely damaged. The findings confirmed that the probe-before-destroy approach, originally suggested for probing the atomic protein structure, also works for the metal electronic structure. However, recent diffraction and spectroscopy data using XFEL pulses with very tight foci, ranging from sub-*μ*m to a few *μ*m and with similar peak power, have indicated that depending on the exact experimental parameters various mechanisms triggered by such intensities can potentially have an impact on experimental observables within the typical XFEL pulse durations of 10–40 fs.^47–55^

In our extensive Mn Kβ XES studies of the OEC using XFELs starting in 2012,^28,36,37,39,40,42,45,56,57^ we have employed PS II samples in solutions and microcrystalline form using various preparations and self-amplified spontaneous emission (SASE) XFEL pulses varying by two orders of magnitude in dose and peak power. Our study reported here addresses to what extent these parameters modify the measured Mn Kβ_1,3_ XES spectra, and how these modifications compare to those corresponding to electronic structure changes of the OEC throughout the Kok cycle. Our study focuses on the comparison of XES spectra taken with different XFEL pulse intensities caused by stochastic shot-to-shot fluctuations. We compare data sets taken at different experimental runs and with different XFEL parameters and sample preparations. The case study we report here provides important information regarding the choice of experimental conditions for the application of x-ray spectroscopy to metalloenzyme studies at XFELs, while understanding the effect of intense XFEL pulses to the x-ray emission spectroscopy data of these systems.

## II. MATERIALS AND METHODS

Details on experimental setup and data analysis have been described elsewhere,^39,40,57^ and the most relevant aspects are summarized here. Samples of PS II solutions and microcrystals were measured using a drop-on-tape setup, with up to three laser flashes for advancing the sample to the desired flash states ranging from zero flash (0F, dark) to three flashes (3F). For details on the illumination parameters, see Ref. 40. Note that the conversion of flash states to Kok cycle S-states involves a deconvolution that we did not perform here, as it introduces noise to the data and is not relevant for the question of x-ray-induced effects we discuss here. Mn Kβ_1,3_ XES was measured using a 16-crystal von Hamos spectrometer^5,31,58^ providing a dispersive line focus on either an ePix100^59^ or Jungfrau^60^ 2D detector situated under/sideways from the interaction point (depending on the XFEL polarization). These detectors vary in pixel size (50 *μ*m for ePix100 and 75 *μ*m for Jungfrau) and thus have slightly different resolution. Data from seven experimental runs at the Linac Coherent Light Source (LCLS)^20^ at SLAC National Accelerator Laboratory was used for our study. This consisted of measurements at the macromolecular femtosecond crystallography (MFX)^61^ beamline with microcrystal samples for four runs and solution samples for one run, and the x-ray pump probe (XPP) beamline^62^ for two solution measurements. Calibration of the spectrometer setup used aqueous solutions of MnCl_2_ as a standard.

SASE XFEL pulses with a photon energy of ∼9.5 keV and an estimated pulse duration of ∼35 fs were used to create the 1s core-hole initial state for our Kβ XES experiments. The beam was focused using beryllium lenses, with the focus ranging from 2.5 to 10 *μ*m full width half maximum (FWHM). To estimate the intensity and dose, we approximate the beam with a circular two-dimensional Gaussian distribution, which contains 50% of the pulse energy—see the supplementary material^71^ Fig. S1 for an illustration of the fraction of the beam falling within the focus. The intensity is then given by 50% of the pulse energy divided by the pulse duration and the area of the focus (FWHM). Similarly, the dose is calculated using this area and the sample length along the beam to obtain the irradiated volume/mass (see footnotes in Table I). The pulse energy was measured upstream of the beamline optics with a gas monitor detector (GMD) and recorded in units of mJ, where 1 mJ = 6.54 × 10^11^ photons at 9.55 keV photon energy. The throughput of pulse energy to the sample is estimated as 0.55 for MFX and 0.60 for XPP and we use these values for the conversion of pulse energy to intensity and dose at the sample.

**FIG. 1.**
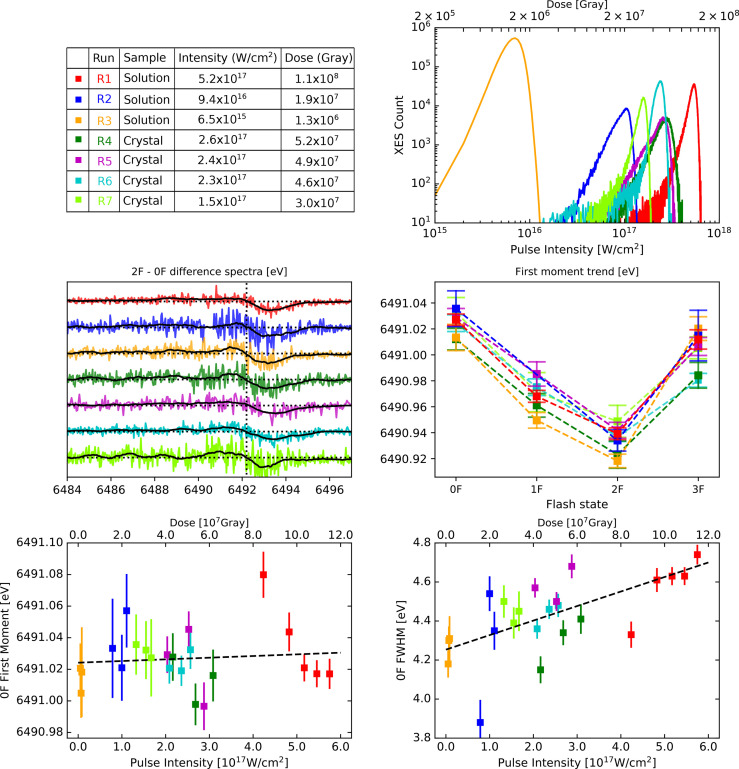
Summary of overall results from all runs (R1–R7), color-coded as per the upper left panel. Top left: Experimental parameters. Top right: histogram of overall summed XES counts as a function of pulse intensity. Middle left: 2F–0F difference spectra, including lines for zero levels (horizontal dotted lines), as well as the approximate inflection point (vertical dotted line). Middle right: first moments for all flash states (0F to 3F). Bottom: first moment (left) and FWHM (right) of the 0F data as a function of pulse intensity/dose, with each run subdivided into five (R1) or three (R2–R7) categories according to different pulse intensity/dose groups (see Figs. 3–5 top left panels). Including linear fits to FWHM and first moment, with R^2^ correlation coefficients of 0.39 for the FWHM, and 0.01 for the first moments.

**TABLE I.** Beam parameters of PS II experiments (R1–R3 solution samples and R4–R7 crystals). Estimated pulse duration is ∼35 fs for all experiments, and SASE XFEL pulses are at 9.5–9.6 keV photon energy.

Exp. run	Pulse energy^a^ (mJ)	Photons per pulse^b^	Beamline throughput^c^	Focus diameter^d^ (*μ*m)	Pulse intensity on sample^e^ (W/cm^2^)	Pulse dose on sample^f^ (gray)	Photon density on sample^g^ (cm^−2^)	Number of photons/pulse absorbed in each Mn atom^h^
R1	3.0 ± 0.3	2.0 × 10^12^	0.60	2.5	5.2 × 10^17^	1.1 × 10^8^	1.2 × 10^19^	0.188
R2	1.5 ± 0.3	9.8 × 10^11^	0.55	4	9.4 × 10^16^	1.9 × 10^7^	2.2 × 10^18^	0.035
R3	0.6 ± 0.1	3.9 × 10^11^	0.60	10	6.5 × 10^15^	1.3 × 10^6^	1.5 × 10^17^	0.0024
R4	4.1 ± 0.7	2.7 × 10^12^	0.55	4	2.6 × 10^17^	5.2 × 10^7^	5.9 × 10^18^	0.093
R5	3.9 ± 0.9	2.6 × 10^12^	0.55	4	2.4 × 10^17^	4.9 × 10^7^	5.6 × 10^18^	0.088
R6	3.7 ± 0.3	2.4 × 10^12^	0.55	4	2.3 × 10^17^	4.6 × 10^7^	5.3 × 10^18^	0.083
R7	3.8 ± 0.4	2.5 × 10^12^	0.55	5	1.5 × 10^17^	3.0 × 10^7^	3.5 × 10^18^	0.055

^a^ Center-of-mass of pulse energy detected by the gas monitor detector (GMD), with spread from standard deviations.

^b^ At 9.55 keV photon energy a 1 mJ pulse energy corresponds to 6.54 × 10^11^ photons/pulse.

^c^ Estimated fraction of pulse energy reaching the sample after focusing and beam transport.

^d^ Estimated diameter of the focused beam on the sample (FWHM) as provided by XPP/MFX instrument settings.

^e^ Estimated pulse intensity reaching the sample, calculated as

$I=\left(\varepsilon \times \alpha \times \beta \right)/\left(A\times t\right),$ where ε is the pulse energy, α the throughput, β the fraction of the beam in the focal region, A the area of the focus (*A* = diameter^2^ * π/4), and t the pulse duration. The focus is estimated to be a circular Gaussian with the area falling under the FWHM counted as the focal region, with 0.5 of the total beam falling within this area (see Fig. S1).

^f^ Estimated dose (Gray = Joule/kg), calculated as

$D=\left(\varepsilon \times \alpha \times \beta \times \zeta \right)/\left(\rho \times A\times l\right),$ where ε is the pulse energy, α the throughput, β the fraction of the beam in the focal region, ζ the fraction of the beam absorbed, ρ the density of water, A the focal area, and l the sample thickness. This is calculated by assuming that the sample is close to pure water and thin compared to its attenuation length (1710 *μ*m at 9.55 keV). In this thin-sample limit, the absorption is linear with sample thickness and the dose is independent of the sample thickness. To calculate the doses, we use a sample thickness l = 100 μm corresponding to an absorbed fraction of ζ = 5.69% at 9.55 keV photon energy.

^g^ Photon density is the number of photons per pulse per unit area that reach the sample. It is defined as

$PD=\left(\varepsilon \times \eta \times \alpha \times \beta \right)/A,$ where ε is the pulse energy, η the conversion factor from pulse energy to photon count (6.54 × 10^11^ photons/mJ at 9.55 keV), α the throughput, β the fraction of the beam in the focal region, and A the area of the focus.

^h^ The estimated number *N* of photons/pulse absorbed by each Mn atom in the focus of the pulse is calculated by multiplying the Mn photo-absorption cross section with the photon density,

N=σ×PD, where σ = 1.57 × 10^−20^ cm^2^ is the photoabsorption cross-section for Mn at 9.55 keV (Ref. 70) and the photon density *PD* is defined above.

To account for charge sharing, which frequently occurs for detectors with small pixel sizes,^63^ the recorded data were corrected for detector artifacts and reduced according to established protocols.^57^ The corrected data were then sorted for sample hits, employing thresholds for the number of photon hits in the region of interest (ROI), as well as per unit area inside the ROI vs outside. The parameters were selected as 3/2.0 when using an epix detector, and 1/1.0 when using a Jungfrau detector. A previous study suggests that this introduces no discernible artifacts, but it improves the signal-to-noise ratio.^57^ Images were further sorted according to pulse energy, as provided by the GMD.

**FIG. 2.**
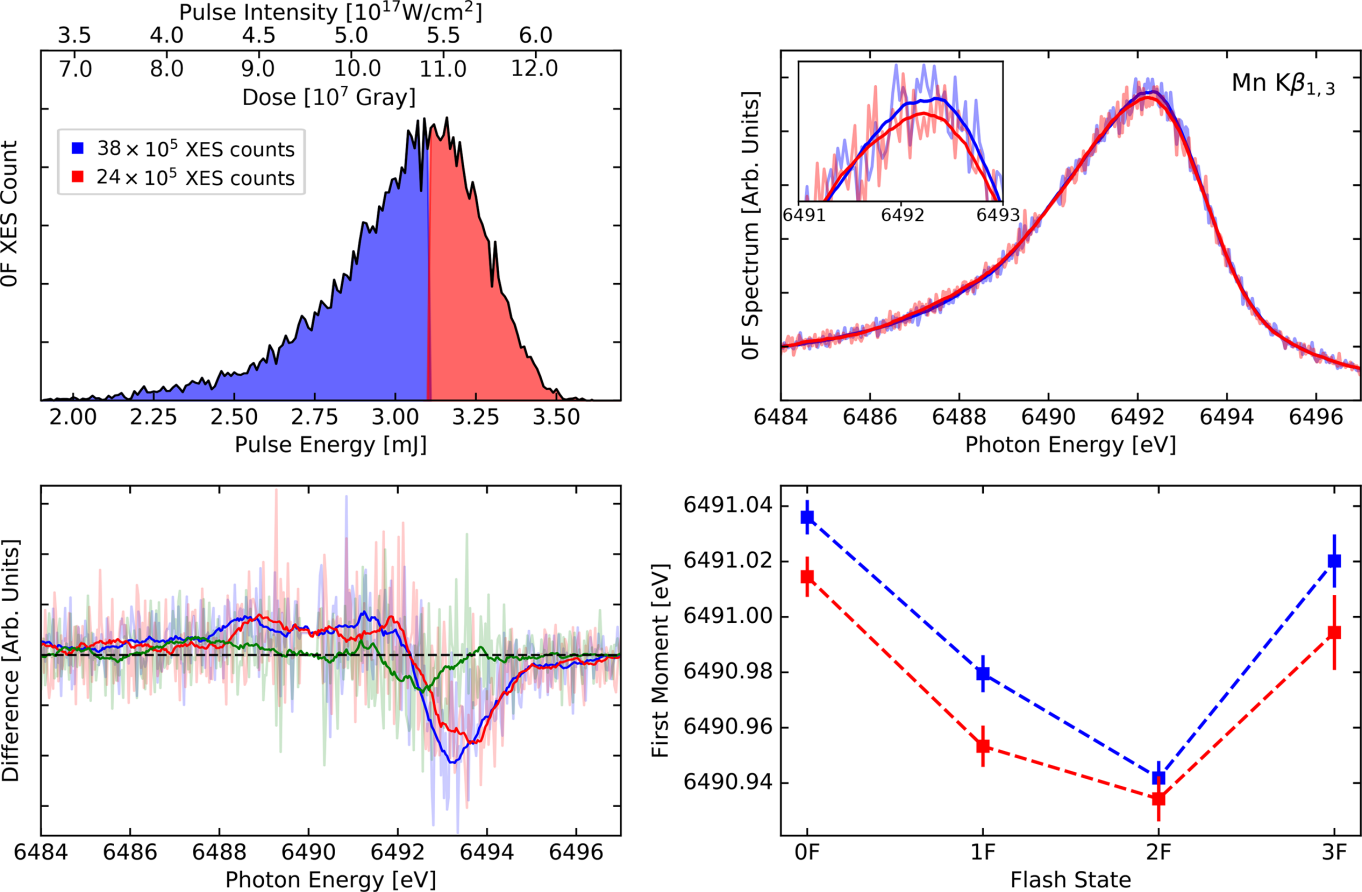
Comparison of data sorted by weak and strong pulses of R1, color-coded blue and red, respectively. Top left: histogram of XES counts of the 0F spectra as a function of pulse energy. (The corresponding pulse intensity shown on the top axis.) We divide the shots into weak (up to 3.1 mJ) and strong (above 3.1 mJ). Top right: area-normalized 0F spectra (smooth and raw). Bottom left: 2F–0F difference spectra for weak (blue) and strong (red) pulses compared to the strong minus weak 0F difference spectrum (green). Bottom right: comparison of first moments for all flash states (0F to 3F) for weak and strong shots.

**FIG. 3.**
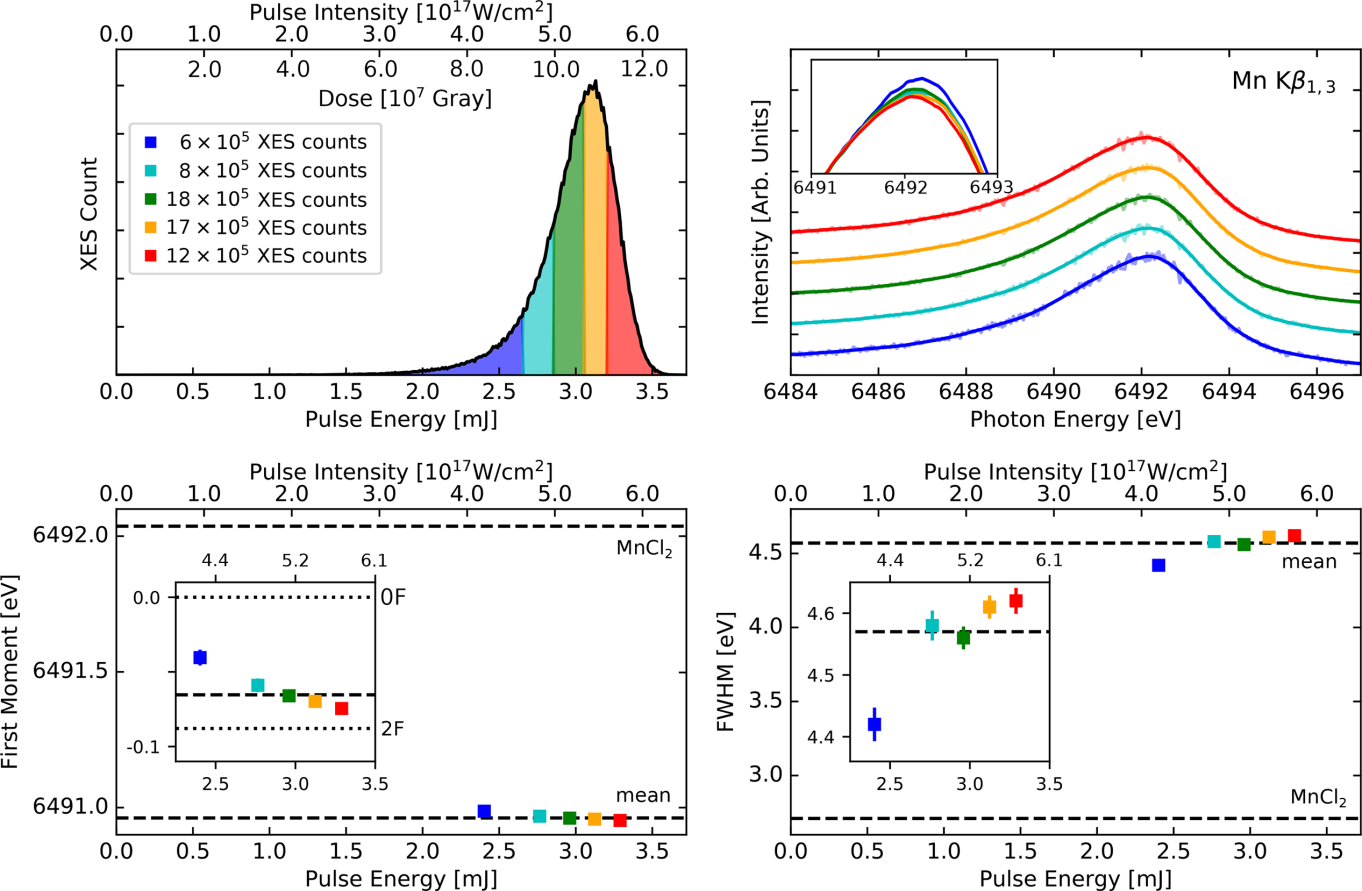
Comparison of data averaged over all flash states sorted from weak to strong pulses of R1, color-coded as per the upper left panel. Top left: histogram of XES counts as a function of pulse energy/intensity showing our grouping into five different regimes. Top right: smooth spectra corresponding to the five pulse energy regimes. Bottom panel: first moments (left), and FWHM values (right) shown as a function of mean pulse intensity of each regime. Mean values over total data sets and MnCl_2_ values are shown for comparison as horizontal dashed lines. Insets show zoomed-in perspectives, and the first moment inset also shows the 0F and 2F results as dotted lines for comparison.

**FIG. 4.**
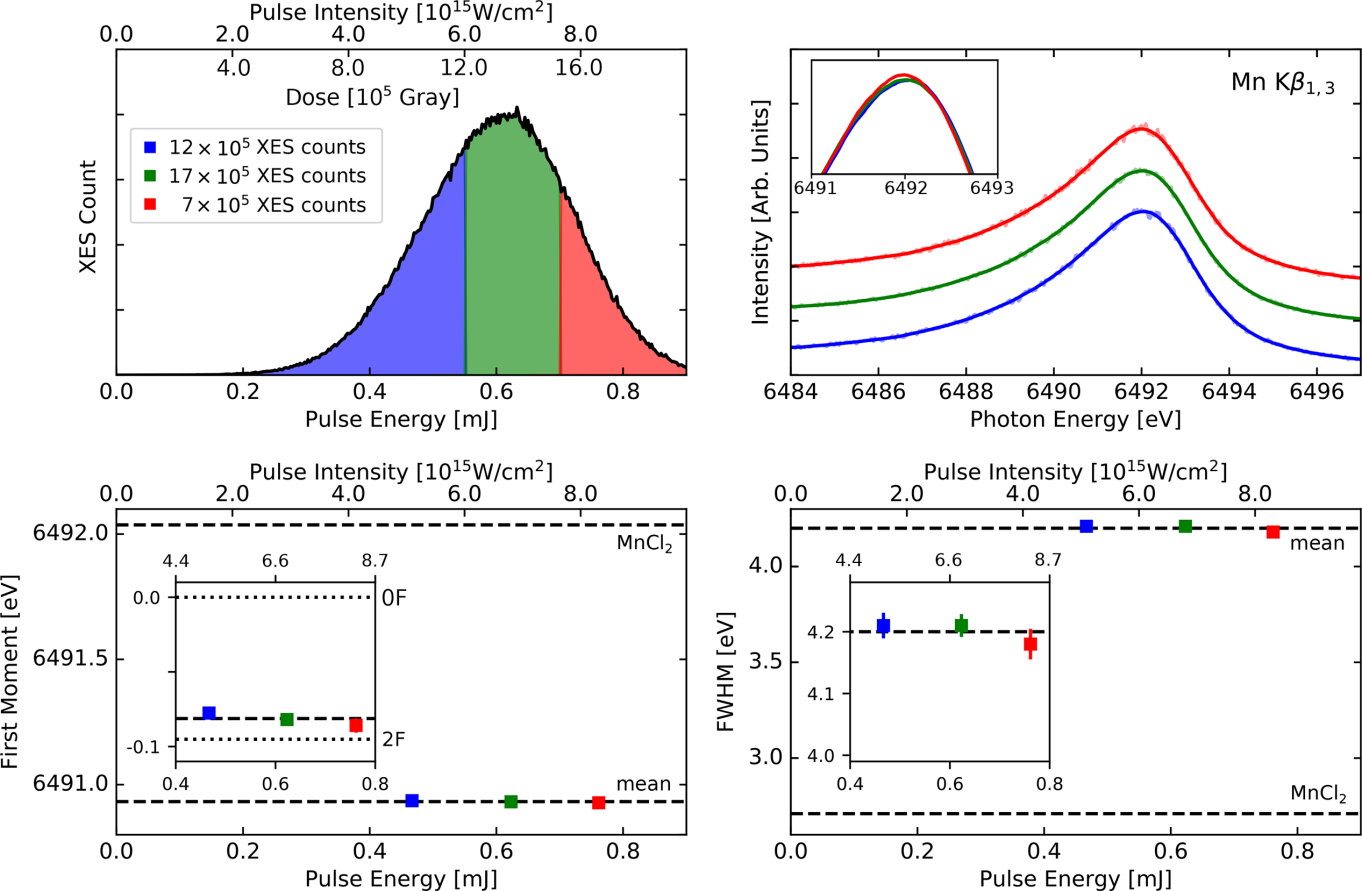
Comparison of data averaged over all flash states sorted from weak to strong pulses of R3 (solution sample), color-coded as per the upper left panel. Top left: histogram of XES counts as a function of pulse energy/intensity showing our grouping into three different regimes. Top right: smooth spectra corresponding to the three pulse energy regimes. Bottom panel: first moments (left), and FWHM values (right) shown as a function of mean pulse intensity of each regime. Mean values over total data sets and MnCl_2_ values are shown as horizontal dashed lines. Insets show zoomed-in perspectives, and the first moment inset also shows the 0F and 2F results as dotted lines.

**FIG. 5.**
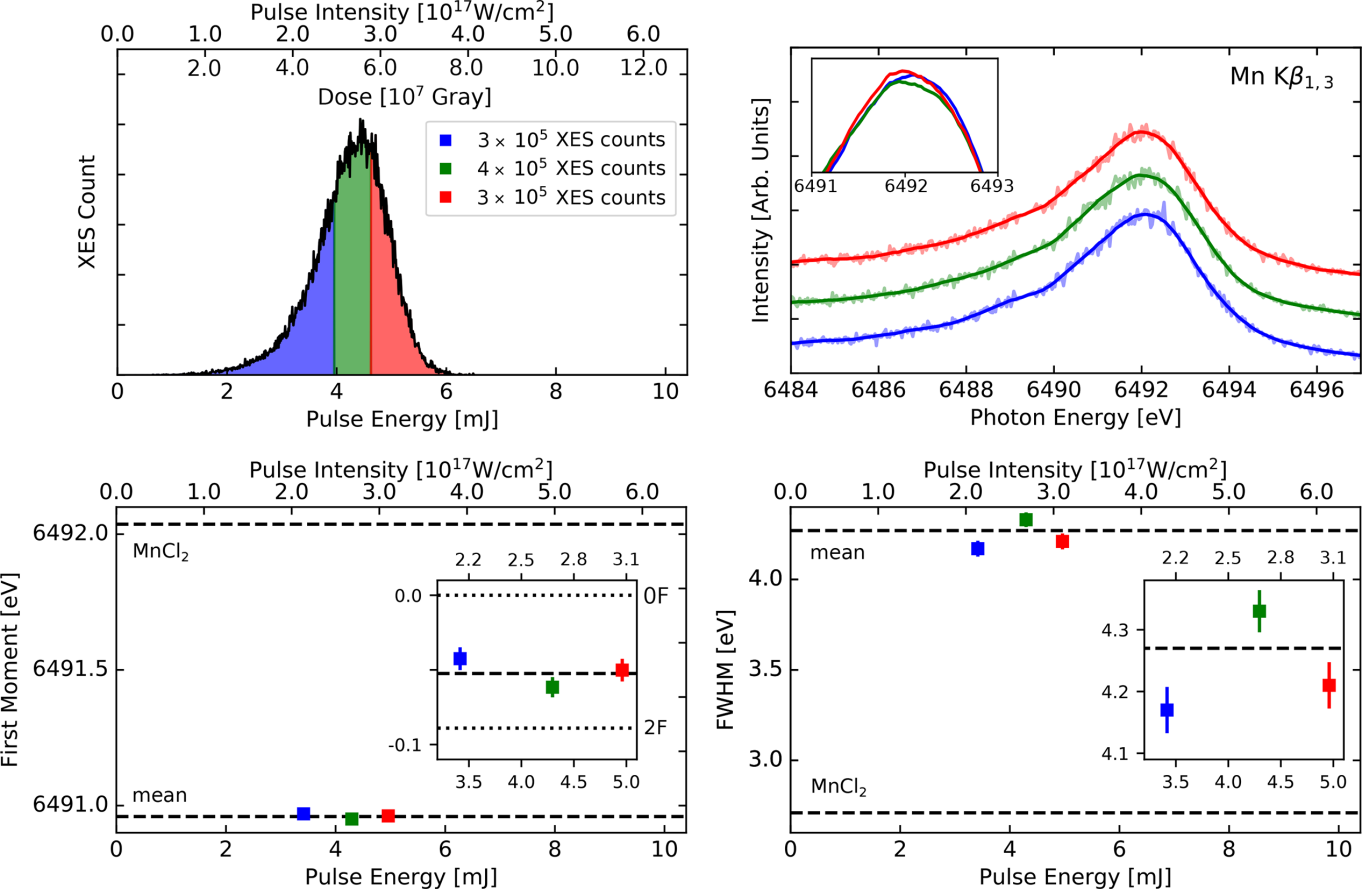
Comparison of data averaged over all flash states sorted from weak to strong pulses of R4 (microcrystal sample), color-coded as per the upper left panel. Top left: histogram of XES counts as a function of pulse energy/intensity showing our grouping into three different regimes. Top right: smooth spectra corresponding to the three pulse energy regimes. Bottom panel: first moments (left), and FWHM values (right) shown as a function of mean pulse intensity of each regime. Mean values over total data sets and MnCl_2_ values are shown as horizontal dashed lines. Insets show zoomed-in perspectives, and the first moment inset also shows the 0F and 2F results as dotted lines.

Spectra are presented in raw format or smoothed using a third-order Savitzky–Golay filter of length 51 (with energy intervals over one pixel ranging from about 0.03 to 0.05 eV, depending on detector), and in both cases area-normalized over 6484 to 6497 eV. The smooth spectra were used for estimating peak width (FWHM), while the first moments were calculated on spectra splined to a resolution of 0.01 eV. The first moment measures the spectral center of mass and is thus primarily used to quantify small overall red and blue shifts. We used an interval of 6485.5–6495.5 eV around the Kβ_1,3_ peak, where the peak height is approximately equal. We note that reported first moments and first moment shifts of Mn Kβ_1,3_ spectra vary in the literature^18,64–68^ due to differences in absolute energy calibration, background subtraction, spectrometer resolution and first moment integration range. Error bars for first moments and FWHM were estimated using photon statistics, with the spreads obtained using a previously established bootstrap method.^57^ We note that other descriptors, such as integrated absolute differences or first moment with variable energy intervals, have been shown to provide similar information as the first moments used here.^69^ Histograms in Figs. 2–5 and in the supplementary material^71^ were constructed using a bin size of 0.0025 mJ for experiment R3, and 0.01 mJ for all other experiments (smaller bins were selected for R3 as the intensity is up to two orders of magnitude smaller). The binning of spectra by pulse energy for any given run is equivalent to binning by intensity, as both the beam focus and pulse duration do not change in a run. For the histograms in Fig. 1, a common bin size of 5 × 10^14^ W/cm^2^ was used. Photon counts are provided for the raw spectra (i.e., with background).

## III. RESULTS

This study comprises data collected over seven different beamtime runs at LCLS. Three experiments used PS II solutions (∼0.8 mM Mn concentration) and four experiments used PS II microcrystals (∼1.0–1.2 mM Mn concentration), suspended in a buffer solution. We order the experimental runs by sample type (solution: R1–R3; microcrystal: R4–R7) and respective beam intensities used in the measurements. Two solution experiments (R1 and R3) were performed at the XPP instrument and R2 at the MFX instrument. All microcrystal experiments were performed at MFX, with R5 being the only one for which we employed a Jungfrau detector (with resulting lower resolution of about 0.05 eV per pixel). Our analysis focuses on the solution experiments with the strongest (R1) and weakest (R3) XFEL beam intensities, and the microcrystal experiment with the strongest (R4) XFEL beam. Sample properties and estimated beam parameters are compiled in Table I. The pulse intensities at the sample and the absorbed doses are estimates, derived from our knowledge of the pulse energy, focus, pulse duration, and beamline throughput downstream of the GMD. It is important to note that the pulse intensity and dose in our experiments track each other linearly, as all of our experiments were performed with x-ray optically thin samples, the same pulse lengths (∼35 fs) and at the same photon energies. We will henceforth focus on the pulse intensity on sample when discussing our results, while providing the corresponding dose in the table and figures.

### A. Comparison of data from all runs

Our overall results for all seven experimental runs (R1–R7) with pulse intensities spanning two orders of magnitude are summarized in Fig. 1. We report the summed Kβ_1,3_ XES signal counts for all experimental runs and information on sample type and estimated pulse intensities (top panels); the 2F–0F difference spectra and first moment values of all flash states (0F–3F) (middle panels); and the Kβ_1,3_ first moments and peak widths of the 0F data collected from each run as a function of pulse intensity (bottom panels). Our selection of 2F–0F difference spectra is motivated by the fact that these states exhibit a large spectral difference. In the bottom panels, we separated each run into three or five different groups according to the beam intensity (see more details below). Illustrations showing the peak width as a function of pulse intensity also for 2F data and when combining all data—regardless of flash state—is found in Fig. S2, and we note that stronger correlation coefficients between pulse intensity and FWHM are found for these data sets (0.57 and 0.72, as compared to 0.39 for the 0F data). This is likely an effect of improved overall signal quality—see discussion below.

### B. Spectral trends observed in the run with the highest pulse intensities

In Fig. 2, we show the spectra, beam intensity histogram, and first moment trends of R1, the run with the highest pulse intensities and doses. We sorted the data set into two groups, weak (A; blue) and strong (B; red), according to the GMD pulse energy values (top left). The histograms are constructed for 0F XES photons, and the total number of photons in each selection is provided in the panel. For clarity, both the pulse intensity and dose are included in the histogram panels in this and all subsequent figures. The top right panel depicts the area-normalized raw and smoothed 0F spectra from the two sets, showing a slight decrease in peak intensity corresponding to a small spectral broadening for B. The lower left panel reports the 2F–0F difference spectra for groups A and B, respectively, and the difference spectrum of the B–A 0F spectra. The lower right panel shows the first moment trend for the flash states, illustrating a general downward shift of up to about 0.02 eV for the B selection compared to the A. The error bar was estimated from bootstrap sampling of the full set of XFEL pulses and thus probes primarily photon noise.^57^ More systematic changes are thus not included in this estimate.

To further quantify the effects induced by intense pulses, Fig. 3 shows the full spectra, first moment, and peak width (FWHM) of five intensity selections for the R1 data averaging over all flash states. We provide similar figures for all other experiments, with the R3 and R4 data in the main text and the rest in the supplementary material (Figs. S3–S6).^71^ Note that the first moment and FWHM error bars can be smaller than the marker size, especially for the zoomed-out perspectives. We further remark that the distribution of measured flash states differs between the experiments, as can be seen by the varying placement of the mean first moments as compared to the 0F and 2F values. In Fig. 3 we observe a consistent—albeit small—trend from weaker to stronger pulses, with a monotonic decrease in the first moment and a more sudden increase in peak width. To put these changes in context with those corresponding to a different chemical environment, we plot the average first moments and FWHM values together with those for aqueous solutions of MnCl_2_ (here 6492.04 and 2.71 eV, respectively) shown as horizontal dashed lines. For the first moment insert we also provide 0F and 2F results as horizontal dotted lines. The five intensity intervals have average beam intensities ranging from 2.40 to 3.29 mJ, corresponding to pulse intensity estimates ranging from 4.19 × 10^17^ to 5.74 × 10^17^ W/cm^2^. The shift in first moments and FWHM between the weakest and strongest intensity selections amounts to −0.03 and 0.20 eV, respectively. The former can be compared to the ∼0.09 eV first moment shift between the 0F and 2F data of R1.

### C. Spectral trends observed for lower pulse intensities

In Fig. 4, we report the results of intensity sorting for solution samples (R3), where LCLS was operated at low intensity (∼0.6 mJ) and with a less focused beam (∼10 *μ*m FWHM) resulting in a pulse intensity that is approximately two orders of magnitude weaker than for R1 (see Table I and upper pulse intensity axes). In Fig. 5, we provide the results for the microcrystal experiment with the highest pulse intensity (R4). The results for the remaining experiments are provided in the supplementary material (Figs. S3–S6).^71^

## IV. DISCUSSION

From Fig. 1, it is clear that we observe very similar difference spectra and first moment trends for the seven experimental runs, with XFEL pulses that vary in pulse intensity by up to two orders of magnitude. These experiments have been conducted over a span of four years, using different experimental stations, samples, sample delivery systems, laser set-ups, detectors, and other parameters. The highly reproducible first moment trends and difference spectra are thus proof of a robust experimental protocol.

A comparison between data collected at LCLS and SACLA x-ray free-electron laser in Japan was included in Ref. 45, focusing on the Mn Kβ_1,3_ x-ray emission spectra of MnCl_2_ and 2F PS (microcrystal) samples. The SACLA data were collected using a weak beam (∼0.3–0.4 mJ) with a significantly shorter pulse duration (∼7 fs) and a tight focus (∼2 *μ*m), leading to approximately the same pulse intensity as R4–R7, but a five times lower dose of <10^7^ Gray (because of the 5 times shorter pulse duration). Within the limited photon statistics, the SACLA spectra were very similar to those from R4–R7, indicating that the pulse length does not affect the spectra at these intensities. This is consistent with the results from Alonso-Mori *et al.*, where only small differences between 10 and 30 fs pulse durations were noted for dilute iron samples.^53^

With regard to radiation-induced effects on the electronic structure, Fig. 2 shows distinct trends of spectrum broadening with higher intensities, as has previously been reported for iron samples,^51,53^ and was also apparent (although originally not noted) in the first XFEL-based XES studies of Mn_2_^III,IV^ Terpy (see Fig. 3 in Ref. 31). These trends are less clear when comparing the 0F peak widths of all experiments (Fig. 1, lower right), and no real correlation is found for the first moment (lower left). Peak widths depend on the exact experimental conditions, which can change between the runs due to slight differences in spectrometer alignment, geometry and resolution, sample conditions, background removal, shielding, and other parameters. We observe such small differences in our calibration runs with MnCl_2_, which varies by up to ∼0.2 eV in FWHM. It is therefore not surprising that we observe relatively large fluctuations in peak widths. A linear fit between FWHM and pulse intensity yields a correlation coefficient of 0.39 for the 0F data in Fig. 1, which increases to 0.57 when considering 2F data and 0.72 when combining all flash states (see Fig. S2 in the supplementary material^71^). The stronger correlation is partially due to better photon statistics. This analysis yields a ∼10% increase in peak width when increasing the pulse intensity by about two orders of magnitude (Figs. 1 and S2). These XFEL-induced effects on spectra are of a different nature than synchrotron radiation-induced electronic structure changes, which instead lead to photoreduction of metals with a corresponding shift to higher energy in XES. The S-state averaging can be done for the FWHM because of its relatively small dependence on the probed S-state. However, comparing first moments of averaged S-states between different experiments can be misleading, because each experiment has a different S-state distribution (see Fig. 1, lower left). Therefore, such a comparison was not performed.

Looking more closely into groupings of data collected within the same experimental runs, we note that only R1 shows clear signs of radiation-induced effects, with distinct increases in FWHM and decreases in first moments. The other experiments have more varying trends, often within the estimated error bars of the FWHM and first moments. There are likely three reasons for these lack of clear effects for these other experiments: (i) the radiation-induced effects are not purely linear with respect to pulse intensity, and will thus become more and more apparent as the beam becomes stronger, (ii) the absolute intensity fluctuations are generally larger for high intensities, and we thus probe a wider intensity distribution, and (iii) the increasing contribution of photon noise with weaker beam intensity, with R1 containing the highest number of XES counts. As such, the remainder of this discussion will focus on the R1 data.

A broadening of spectral features upon XFEL-induced electronic structural changes agrees with previous studies on radiation-induced effects of Fe XES,^51,53^ as studied in detail for Kɑ and Kβ emission spectra of both iron foils and solutions.^53^ These broadenings were described as indicators of radiation-induced effects on the electronic structure due to redistribution (removal) of electrons in the vicinity of the probed atoms. A strong dependence of these effects on iron concentration was observed for high concentrations, but not at low concentrations. This can be understood by the fact that below 450 mM concentration the absorption from the solvent starts to dominate. Consequently, well below this threshold, the effects of the released electrons on the Fe XES signal become concentration independent.

Our PS II samples should thus not exhibit any concentration dependence in radiation-induced effects (although it can be noted that the limit is likely somewhat lower than for the Fe samples, as the oxygen-evolving complex possesses four Mn atoms in relatively close proximity). In terms of quantifying the electronic structure effects of our measurements, when applying the rate equation model from Ref. 52 and adopting a 1s photoelectric cross section of ∼1.5 × 10^−20^ cm^2^, we obtain an α value of 0.18 for R1. This parameter is formulated to encapsulate most of the pulse effects and allow for easy comparison between experiments. A value of 0.18 would correspond to ∼6% of the total emission signal being non-classical or originating from sequentially ionized atoms. Using the same model for our microcrystal experiment with the highest pulse intensity (R4), we obtain an estimated ∼3% of total emission being non-classical for that experiment. The effect on the atomic structure due to this relatively small change in electronic structure is unknown, but changes at a 3% level would likely not be visible in the XRD analysis. A different measure of the amount of non-classically emitting sites can be obtained by considering the number of photons absorbed per Mn atom ranging from 0.0024 (R3) to 0.188 (R1) (see Table I). Assuming that the absorption is dominated by single-photon events, we use these values as the probability of single-photon absorption by the Mn atoms in each run. With this, we can calculate the probability that more than one of the four Mn atoms in the OEC absorbs a photon. The resulting values are 0.16 for the highest intensity (R1) and range from 0.02–0.05 for the microcrystal experiments (R4–R7).

From Figs. 2 and 3, we note that moving from weak to strong beams yields a progressive broadening of the spectral features, resulting in changes in peak width and first moment of 0.20 and −0.03 eV, respectively. Difference spectra are only weakly affected, with 2F–0F of the strong pulses being slightly less pronounced, and the 0F strong–weak difference mainly showing a negative feature around the peak maximum (see Fig. 2, bottom left). Furthermore, the first moment trends in Fig. 2 show a general decrease in about 0.02 eV in first moments throughout the Kok cycle, being less pronounced for the 2F data. We note that the Mn oxidation states throughout the metastable intermediates vary from [III_3_,IV] (S_0_) to [IV_4_] (S_3_). With the dark stable state being S_1_, 2F data are thus dominated by the most highly oxidized S_3_ state. As such, 2F–0F difference spectra show the largest changes, which is why we focus on these differences for direct spectrum comparisons. We speculate that the less pronounced change in 2F first moments may be due to the fact that the 2F state has the highest Mn oxidation state (i.e., least number of valence electrons) which makes it less likely to remove more valence electrons. It can also be related to the flattening of trend series that occur when the tails/background levels are increased—as the underlying pedestal is increased in intensity, all first moments trends move toward the middle of the adopted energy interval.^57^ Importantly, we find that the first moment shifts even at the highest pulse intensity are relatively small compared to the flash state shifts, and that the flash state shifts remain very similar throughout all pulse intensities. Thus, time-resolved studies of spectral trends are reliable, even when small radiation-induced effects are present. To minimize the small radiation-induced effects that we do observe, all flash states should be measured within a similar range of pulse intensities.

## IV. CONCLUSIONS

We have studied the effects of pulse intensity/dose on Mn Kβ_1,3_ XES spectra from microcrystal and solution samples of PS II collected over seven XFEL beamtimes. The experiments were performed at LCLS using a nominal beam energy of 9.5 keV, a pulse duration of ∼35 fs, and beam focus of 2.5–10 *μ*m corresponding to pulse intensities ranging from 6.6 × 10^15^ to 5.7 × 10^17^ W/cm^2^ and doses ranging from 1.3 × 10^6^ to 1.1 × 10^8^ Gray. Considering each experiment individually, we only find evidence for x-ray induced spectral effects for the solution experiment performed at the highest pulse intensities, yielding a small spectral broadening of the Kβ_1,3_ peak and first moment shift to lower energy, which both increase with pulse intensity. Considering all experiments, we observe an overall trend of intensity-related increase in peak widths of ∼10% when comparing the spectra collected for the strongest vs weakest pulse intensities. Our observed spectral broadening is consistent with recent results obtained for iron samples^53^ where a strong dependence on sample concentration was reported for samples with two orders of magnitude higher concentrations. Importantly, we observe that first moment shifts reflecting the small electronic structure effects of the different flash states of PS II are comparable for all pulse intensities, with some slight flattening observed when using the most intense beams. This shows that intensity-dependent effects on the Mn Kβ_1,3_ XES have very little impact on observing the subtle chemical changes in the photosynthetic cycle of PS II. Our study thus supports the findings of time-resolved studies on PS II carried out with the various beam intensities.^40,42,45^ While future studies with more sensitive spectral probes might yield an enhanced sensitivity to beam-induced effects, we can cautiously project our findings to other studies of metal centers in other dilute metalloproteins. The data presented here suggest that at 9.5 keV photon energy, 3 mJ pulse energy, 60% beamline throughput, 3 *μ*m diameter focus (FWHM), and 35 fs pulse length, the corresponding pulse intensity of 3.6 × 10^17^ W/cm^2^ and dose of 7.4 × 10^7^ Gray is unlikely to cause any appreciable effects on the electronic structure measured by Kβ XES. However, more studies are needed to establish the effects on valence-to-core XES^53^ and other more directly valence-sensitive spectroscopies.

## Data Availability

The data that support the findings of this study are available within the article and its supplementary material.^71^ The data that support the findings of this study are available from the corresponding authors upon reasonable request.
